# “When I think of mental healthcare, I think of no care.” Mental Health Services as a Vital Component of Prenatal Care for Black Women

**DOI:** 10.1007/s10995-021-03226-z

**Published:** 2021-09-14

**Authors:** Shakkaura Kemet, Yihui Yang, Onouwem Nseyo, Felicha Bell, Anastasia Yinpa-ala Gordon, Markita Mays, Melinda Fowler, Andrea Jackson

**Affiliations:** 1 University of California San Francisco School of Medicine, 505 Parnassus Ave, San Francisco, CA 94143 USA; 2 Chinatown Public Health Center (a Member of San Francisco Health Network), 1490 Mason St, San Francisco, CA 94133 USA; 3 East Bay Sutter Health OBGYN Group, 2815 Telegraph Ave, #200, Berkeley, CA 94705 USA; 4 Black Infant Health, 1290 Fillmore, Suite 103, San Francisco, CA 94115 USA; 5 Bayview YMCA Family Resource Center, 160 Lane Street, San Francisco, CA 94124 USA; 6 UCSF/SFGH Child Trauma Research Program, San Francisco, USA; 7 Department of Obstetrics, Gynecology, and Reproductive Sciences, University of California San Francisco, 2356 Sutter Street, J-140, San Francisco, CA 94143 USA

**Keywords:** Group prenatal care, Maternal health, Mental health, Maternal health disparities, Racial concordance

## Abstract

**Purpose:**

Black people give birth joyously despite disproportionate rates of adverse perinatal outcomes. Given that group prenatal care shows promise in mitigating these inequities, we sought to solicit the opinions of Black peripartum women on how group prenatal care could be tailored to fit their specific needs. In this study, we describe attitudes about a proposed Black group prenatal care in a single focus group of 11 Black women who receive maternal health services from Black Infant Health (BIH, a state and federal funded state-wide program for Black pregnant people with the goal to improve infant and maternal health). These data were used to design a race-conscious group prenatal care curriculum specifically for Black women at UCSF.

**Description:**

This study was an analysis of focus group data generated as part of a larger project focused on community involvement in Black maternal health. English speaking pregnant or recently postpartum women age 18 or older who receive services from BIH were recruited to participated in the focus group analyzed in this study. All facilitators of the focus group were Black women in order to facilitate candid conversation about racism in prenatal care.

**Assessment:**

The need for mental health care was common thread underlying all conversations about prenatal health improvements desired by our focus groups. Participants expressed the centrality of mental health access during our discussion of other themes (e.g.: ease of access, inclusion of partners, special classes for teen moms) by discussing them in terms of their relationship to mental health. Our participants’ clear expression of the centrality of mental health care to their prenatal health guided our decision to focus on mental health as a necessary pillar of any group prenatal care intervention designed to mitigate perinatal healthcare disparities in this paper. Three themes related to mental health integration into group prenatal care emerged from thematic analysis of the transcripts. Participants expressed insufficient access and advocacy, and provider distrust.

**Conclusion:**

Evidence exists supporting group prenatal care as a tool for mitigation of perinatal health disparities among Black women. There is also a large body of data describing the disproportionate burden of mental health needs among Black women. The rich data we present here from Black women on their desire for the integration of these two needs fits well into the parallel conversation occurring in the literature. To our knowledge, this is the first study investigating desires of Black women regarding group prenatal care designed specifically for them. They expressed a strong desire for more access to mental health care providers who are racially conscious and aware of white supremacy, and nuanced opinions on the role of racial concordance in health equity.

## Significance

Black women give birth joyously despite adverse perinatal outcomes such as preterm birth (PTB), small for gestational age (SGA), lower breast-feeding rates, and maternal and neonatal deaths at higher rates than any other racial group, even when matched with women of the same socioeconomic status (Martin et al., 2018). Nationally, the PTB rate for Blacks is 13.93% compared to Non-Hispanic White women for whom the PTB rate is significantly lower (Martin et al., 2018). New widespread interest in the Black Lives Matter Movement has placed renewed attention on perinatal health disparities by race, and the COVID-19 pandemic has unfortunately only added a new dimension to the already-deadly crucible of health inequity, systemic racism, and sexism in which Black pregnant people choose to mother (Egede & Walker, 2020). Multiple modifiable factors have caused racial health disparities in PTB, including psycho-social stress, pregnancy-related anxiety, and nutrition status (McEwen, 2009; McLemore et al., 2018; Schneiderman, Ironson, & Siegel, 2005). Especially given the increased need for mental health support for Black women in the context of COVID-19, comprehensive care that addresses psychosocial determinants of maternal and infant health outcomes are essential to improve birth outcomes (McLemore et al., 2018; Novacek et al., 2020; Vedam et al., 2019).

Group prenatal care has proven effective in improving maternal and perinatal outcomes (Carter et al., 2016; Chen et al., 2017; Cunningham et al., 2019; Ickovics et al., 2007, 2016). This type of clinical care places 8 to 12 pregnant women at similar gestational ages in group sessions. During these sessions, participants assume an active role in their care by measuring their own blood pressure and weight, followed by facilitated group discussions on topics such as nutrition, stress reduction, relationships, and parenting. We convened a single focus group of eleven Black pregnant or recently postpartum women to solicit their feedback on how the group prenatal care model could be best modified to fit their specific needs in the context of the unique perinatal healthcare disparities experienced by Black women.

## Introduction

Results from several peer reviewed studies suggest an association between participation in group prenatal care and improved maternal and infant outcomes, including reduced rates of PTB, increased initiation of breastfeeding, and patient satisfaction. Some data suggest that there may be a unique positive effect for Black women. A meta-analysis of four randomized control trials and ten cohort studies (n = 10,321) comparing group prenatal care with traditional care found no significant difference in rates of PTB or breastfeeding initiation (Carter et al., 2016). However, when analysis was restricted to Black women in high-quality studies there was a statistically significant decrease in PTB (n = 992; pooled RR = 0.55). These data suggest that Black women may derive a unique benefit from group prenatal care that we seek to capitalize upon in order to help close racial maternal health disparities.

The unmet mental health needs of Black pregnant women are well documented. It is estimated that 10–20% of pregnant and recently postpartum women experience depressive symptoms (*Position Statement 49*, n.d.), and that the rates among low-income and Black women are even higher (Mukherjee et al., 2016). One major factor associated with depression that is unique to Black women is the racism they experience before, during, and after pregnancy (Berger & Sarnyai, 2015; Ertel et al., 2012; Williams, 2018). Moreover, the race-based violence and disproportionate effect of COVID-19 on Black people may increase this already disproportionate mental health burden on Black pregnant women (Novacek et al., 2020). Research suggests that depression during pregnancy is a risk factor for adverse neonatal outcomes such as PTB, low birthweight, and SGA (Szegda et al., 2014), and that provider communication about depression in the context of its ethnicity-specific risk factors during the antenatal period provides a protective effect against postpartum depression (Mukherjee et al., 2018). In combination, this evidence suggests that group prenatal care designed to address root causes of perinatal health disparities experienced by Black women must address mental health needs. Furthermore, such care must be done by providers skilled in discussing the effect of racism on health. For this reason, we specifically asked questions about mental health needs in our focus group. We also inquired about the role they thought racially-concordant care should play in group prenatal care for Black pregnant women in light of evidence that racism-conscious and/or racially concordant healthcare can help to mitigate racial healthcare disparities (Alsan, Garrick, & Graziani, 2019; Cuevas, O’Brien, & Saha, 2016; Greenwood et al., 2020; Shen et al., 2018). This focus group was facilitated by self-identified Black women to allow candid responses to these sensitive questions. These facilitators were members of BIH, and no UCSF prenatal care providers were present.

This study was an analysis of focus group data to identify and prioritize aspects of group prenatal care that are important to Black patients. Data from this study was used to design EMBRACE, a racially concordant group prenatal care program that centers the mental health of black birthing people and extends 12 months postpartum (*EMBRACE*, 2020).

## Methods

### Setting and Sample

This study was an analysis of focus group data to identify and prioritize aspects of group prenatal care that are important to Black patients. Participants were recruited from San Francisco BIH Program, an organization funded by the California Department of Public Health to address health disparities affecting Black women and their infants (San Francisco Health Network, n.d.). This organization was chosen due to their close connection to the San Francisco Black community and its ability to provide a study sample representative of the patient population most affected by perinatal health disparities by race, and currently given the least support by the healthcare system. In order to reassure participants that participation in the study would not affect their care at UCSF, BIH staff rather than UCSF clinicians recruited their clients by asking them if they were interested being part of a focus group. They were also provided a sheet that described the purpose if the study, their role as participants, confidentiality, their right to withdraw at any time, and BIH contact information for further questions or concerns. This sheet was again provided as at the time of the focus group as a reminder. The focus group was conducted at the BIH community center by two Black female staff members (FB, AYG), and one Black female UCSF medical student (SK). Eleven BIH clients participated in the focus group. All participants identified as Black birthing people and lived in the San Francisco Bay area. The discussion about group prenatal care began with facilitator explanation of group care and potential benefits for Black women. BIH clients who were 18 years or older and who were pregnant at the time of data collection (n = 9) or had recently given birth to a live infant (n = 2), were invited to participate. Participants were provided $50 gift cards as remuneration for their time. A meal, on-site childcare, and transportation to and from the focus group site were also provided to participants (Table 1).

**Table 1:** Demographics

Demographic	Value	Number of participants (%)
Race	Black	11 (100)
Pregnancy status at time of interview	Pregnant	9 (82)
	Recently (≤ 6 weeks) postpartum	2 (18)

### Interview Guide

We asked open-ended questions about group prenatal care to explore existing feelings about the model before the focus group. AJ and FB wrote the interview guide used in the focus group. Participants were asked about the Finnish baby box intervention (Lee, 2013) in order to start a conversation about global norms and standards about safe infant sleeping. The Finnish baby box is a collection of newborn care items given to parents by the Finish government that has been linked to low infant mortality rates. Then, participants were asked about Centering Pregnancy (*Centering Pregnancy Program*, n.d.) in order to gain insight about past experiences or opinions about group prenatal care. The guide also included questions about mental health and social support, community and support networks, provider preference, and preferred site of care within the UCSF health system (see Interview Guide). Questions about provider preference were included in order to discuss racial concordance. When responding to these questions, participants were most vocal about mental health and social support. When asked questions that were not directly about mental health needs, participants chose to frame their responses in terms of the ways that mental health concerns related to the topic of the question. Their expressed understanding of mental health as foundational to all aspects of prenatal care probed during the focus group prompted the framing of the results in similar terms. We chose to make mental health the focus of this paper to reflect its centrality to our participants.

### Ethical Considerations

This study was reviewed and deemed exempt from human subjects procedures by the University of California, San Francisco Committee on Human Research # 17-22659. Exception was granted because the focus group was part of a quality improvement initiative at UCSF to assess community engagement in a potential prenatal health program. Participants were informed that the purpose of the group was to better understand their attitudes and preferences toward group prenatal care, specifically one that was Black-centered. Participants were also informed that their participation (or lack thereof), as well as any information they shared during the focus group, would not impact the availability and quality of services they receive at BIH or of any ongoing or future care at UCSF.

### Data Collection

The focus group was facilitated by two BIH staff members who are also co-authors on this paper (FB and AYG). SK was also present as an observer and note-taker.

### Analysis

The focus group was audio-recorded and then transcribed verbatim. The first author listened to the recording and reviewed the transcript for accuracy. Three authors (SK, AY, ON) with training in qualitative data individually generated codes based on line-by-line coding. These authors met to resolve discrepancies between their individual coding, as well as to define the major themes and choose salient quotes for each theme. An iterative process was used to refine the themes and quotes, choose exemplar quotes to be included in the manuscript, and complete the report. We followed the COREQ consolidated 32-item checklist for reporting important aspects interviews and focus groups, including methods, context, findings, and analysis.

## Results

Eleven BIH clients participated in the focus group. The discussion about group prenatal care began with facilitator explanation of group care and potential benefits for Black women. Then, the group discussed the features of such a prenatal care group that they would like to see UCSF offer. Immediately, women focused on improved access to mental health care as an urgent need for any group prenatal care designed for their community. When other aspects of prenatal care were discussed, such as continuity of care throughout pregnancy and social support to feel prepared for motherhood, participants expressed that these other components were important because of their potential to improve the mental health of Black pregnant women in their communities.

Participant framing of mental health as crucial to the improvement of prenatal care for Black women is mirrored in our choosing of mental health themes for the results of this paper with quotes that elucidate the connection between mental health support and other aspects of prenatal care as expressed by our participants. The themes discussed at length were lack of access to care, consequent provider distrust, and the need for advocacy for mental health care access to improve. Each theme is described below with quotes that demonstrate the essence of each theme.

### Access

When asked about current barriers to comprehensive prenatal care, many women described situations in which they would report to their provider new mental health concerns during pregnancy or exacerbations of pre-pregnancy mental health issues with their prenatal provider. The provider would then offer mental health referrals that patients found nearly impossible to turn into a mental health appointment:

It's like you talking to the same wall over and over again, it's like you beat me over the head with this wall and nobody's listening.

This participant had managed her anxiety and depression before pregnancy without therapy or medication but found that the added stress of pregnancy worsened her symptoms past the point she could manage alone. Once she expressed to her provider being overwhelmed, she received a referral for counseling from her prenatal provider. This resulted in countless hours on the phone with her government-sponsored insurance representative in an attempt to find a mental health provider that was covered by her insurance, taking on new patients, and had an appointment time that fit her schedule. She was unable to find a provider during her pregnancy and recalled this lack of support as the barrier to access that most negatively impacted her pregnancy.

Another woman also cited the difficulty of working through the prerequisite research and insurance system navigation to see a provider, and explained that it compounded stress in the already-stressful setting of pregnancy. She also mentions the conflict between the perception that Black women fail to reach out for this kind or support and the reality that they do, but often do not receive it.

With MediCal (California government insurance) you have to do all the research yourself, and you're already stressed out, and it's like, it's too many loops...And they wonder why we don't access these services.

Yet a third woman shared that she was able to initiate care, but insurance complications led to the early termination of that care:

I was seeing a lady, but I had issues with my insurance… my therapist wasn't getting paid for our sessions, so I had to stop seeing her. And I didn't know what to do in terms of finding a therapist that my insurance covered... A lot of back-and-forth, you know, calling this person saying to call that person.

Unfortunately, these women were success stories relative to another cohort of women who cited the necessity of a referral to gain access to mental health care as a large barrier. This topic arose when one woman who did not know that she needed a referral expressed her difficulties in trying to get mental health care:

I tried to set an appointment, and the lady is pretty much telling me that because I haven't been seen they can't schedule me a follow-up. They said I had to get another referral?

Even at the time of this focus group, months after the incident had occurred, this participant still did not understand the role that getting a referral played in her care. Several other participants quickly defined the role of a referral in the process for her, but this explanation was never given to her in the many times that she tried to access care. Thus, the participant had gone most of her pregnancy without care.

That participant’s predicament also presented another problem with the referral process: patients must be recognized as mentally distressed by an external standard to access care. The two participants quoted below expressed the sentiment that to receive timely mental health care as a pregnant black woman, they had to present as an imminent danger to themselves or others. They specifically described how they perceived race to affect their mental health care access in pregnancy:

…if I come in here talking to you with some common sense of attitude, calmly, respectfully, and ask ‘can you help me?’ you're just going to send me packing. But if I come in here and just yell and scream and I'm just jumping off the walls acting like I might hurt myself, or somebody else, you're going to get me some help. I don't think that's the way it has to be.

I go [to the mental health provider] when something goes left [goes very wrong]. I'll be yelling and hollering and I can't calm down, I can't slow down. So now you see I'm breaking down in here. What are you guys going to do to help me?

This requirement to present as “jumping off the walls” and “acting like [she] might hurt someone” creates two problems for participants: (1) mental health status must deteriorate to the point of “breaking down” to trigger access, and patients suffer preventable morbidity in the interim, and (2) Black women must behave in a way that reinforces harmful racialized stereotypes such as “the angry black women” or the “savage” to receive care (Ashley, 2014; Walley-Jean, 2009).

The discussion of how severe mental health needs need to be in order to access treatment among Black pregnant women started another about intersecting vulnerabilities that the participants faced. It quickly became evident that the unnavigable nature of the current system disproportionately affects those who might need care the most. Homelessness, food insecurity, and lack of adequate social support made it incredibly difficult for participants to receive mental health care. One participant spoke about her experience seeking mental health care as a pregnant Black woman with one special-needs child for whom she is the main caregiver:

I literally have to take extra steps... I ain't got time for that. I'm dealing with a special needs child, barely got time for myself, just hoping that you could give me some medicine so I could relax for five minutes, please.

Two other women also commented on the challenges of seeking mental health care as a pregnant woman while also battling structural and social determinants of health such as lack of transportation, money, or social support:

I mean like you gotta be there at seven o'clock in the morning, so you can be one of the first people to be seen, and once you go through that, then maybe you can usually make a follow-up appointment, so that you can be seen kinda regularly. But who has time for that? And when your mind is in a bad place, it's kind of hard to get up, to set up a time at seven o'clock in the morning to be seen, then let you know what my problems and issues is, and then have to come back for a follow-up appointment.

So are you telling me I'm seven months pregnant, I'm totally stressed out, I'm crying every day, I'm losing my mind, I can barely hang on… I'm telling you this, and you're telling me I can't be seen? Mind you, it was stressful to call them just to see if I had an appointment that morning. I called for a whole hour. I could barely get through.

When I think of mental healthcare, I think of no care.

When asked how they saw group prenatal care playing into their communities and social support (see Interview Guide: III Community, Kinship, and Support Networks Q1–3) several women said that they needed support to navigate the convoluted mental health care system, bringing up the role of mental health in group prenatal care yet again. Participants shared that this would be particularly helpful for those who are publicly insured:

So outlining, specifically, this is the number to call, this is the services and location of healthcare providers that offer this. I feel like it should be simple.

Maybe if you have MediCal map out like concrete instructions on… seeking mental health.

### Provider Distrust

Patients expressed a general mistrust of the healthcare system throughout the focus group. In the context of a conversation about participants feeling that the healthcare system withholds care from them for racist reasons, a question from the interview guide was asked about potential racial preference for group prenatal care providers (see Interview Guide: IV Provider Preference Q1). A myriad of opinions was brought up by this question, specifically as it applied to mental health concerns. Participants felt that mental health care was different from other aspects of prenatal care in that it required more vulnerability on their part, and expertise on the part of the provider about the intersecting vulnerabilities discussed in the “Access” portion of this paper. However, opinions were mixed on how the race of the mental health provider would play into their care.

Several participants said that mental health providers are motivated by financial considerations rather than quality patient care in a way that they thought was different from other types of healthcare providers. One participant cited her perception that mental health providers were much more willing to prescribe medications to treat her mental health concerns rather than provide talk therapy. She attributed this behavior to profit-seeking behavior of psychiatrists, whom she believes are paid based on the number or medications they prescribe:

And they rush to just throw you medicine. It's like, I could not even need medicine. I might just need somebody to talk to for like an hour, and it's like ... I might just need that good 45 minutes, to just let go. Oh no, no, no, no, no. We going to send you out of here with two prescriptions. But I don't really think I need all of this. I think I might just need someone to talk about things that I build on up in my head. And I really think their paycheck is based on how many medicines they give out.

Participants also perceived evidence of the higher priority placed on the financing of mental health care compared to other forms of care they receive. Moreover, they felt that having public insurance did not get you the same quality care as if you could pay out of pocket for care. They noted that this was different from the rest of prenatal care, which they felt MediCal did give them access to. Given that most participants and their friends were insured through MediCal but still unable to access mental health care, they concluded that one had to not only be insured but also be wealthy to access mental health care.

Yeah, it's pretty much [financial] resources. You can have MediCal all day, but if MediCal don't let you go see someone, then it's pointless to have MediCal.

Participants were concerned that racial concordance without intentionally-antiracist care would not assuage their concerns about receiving subpar mental health. One reason for this was that participants believed that even Black providers have must adopt a the same “white supremacist” viewpoint as White providers in order to be successful in the White-dominated space of psychiatry, and that this mindset would result in worse care for Black patients:

There are a lot of Black mental health professionals out there. There's a lot more than you think, but they so go into this white supremacy realm that they even stop caring about our Black mental health.

I just don't feel like Black, um, mental health is a concern in a white supremacy pyramid.

Throughout this focus group, participants used the term “white supremacy realm/pyramid” to reference their belief that society is structured such that White people are given advantages at the expense of Black people, and that healthcare is designed intentionally to cater to the health outcomes of White people whether or not that structuring helps, harms, or does not apply to Black patients. Thus, their comments express concern that Black providers are so indoctrinated into a society in which Black lives are devalued, that even Black providers are often not able to care about the mental health needs of Black patients merely because they were Black providers. They wanted Black providers who advocated for antiracist care, as discussed in the “Advocacy” section that follows.

When responding directly to the role of racial concordance in prenatal care, women’s responses were complex. Some women immediately said that they would definitely want a Black group prenatal care provider. One postpartum participant had an all-Black provider team during her most recent delivery and reflected, smiling:

I had nine black doctors. I just thought it was everything [was amazing/good]. It was wonderful. Cause that was my first experience [with Black providers] ever, like when I had my daughter everybody was White. Every face was White. Every face, not one Black. It was all white. And then when I had my son too, it was all White. There was no Mexican, no Blacks, no nothing. So for this to be all Blacks I was like, wow. Even in the video you can hear his grandma like, all these beautiful Black nurses and doctors delivering my grandson. Like it was just, you know, shocking, overwhelming, happy thing for us.

Another woman responded quickly:

You're so lucky, I was in the hospital like, I don't want no more White people coming in here.

She continued her thoughts by expressing that after experiencing the White supremacy pyramid in her healthcare to date, she did not want a Black doctor so much as she did not want a White one:

Pilipino. Mexican. Anything but White. Put a sign on the front door.

Another group said what was most important to them was that they had a provider that understood their needs, touching on the idea that racial concordance was desired but not sufficient:

When it comes to dealing with a Black person, you definitely need somebody with that state of mind, or that has been there, or knowing what's actually going on and not just reading the papers and like ‘I learned this in medical school, or I learned about this in my classes’…

One woman expressed the same sentiment with an ambivalence that was characteristic of several responses in the group. Although she said that race did not matter to her, she also said that she wanted a provider who had “living experience” of what she was going through as a Black-pregnant woman. Another woman stated that although she was okay having a provider of any race for herself, she was intent on finding a Black provider for her newborn son.

Finally, without commenting on a preference for one race, another participant shared that she had a White male doctor during her pregnancy who she felt very happy with, and why:

...because he was so cool…he knew about the homeless prenatal shelter, he knew about all the stuff going on, he cared, he was like, do you know about this? Do you know about that? He made sure I knew every resource that was out there…he made sure to tell me about them.

In combination, these quotes suggest that some Black women desire racially concordant care, others believe concordance must come with intentional antiracism, and yet others are happy with care that is antiracist without racial concordance. For our participants, antiracism included knowledge about the ways in which racism affected their pregnancy experience, the ability to discuss it during prenatal visits and birth, and dexterity offering resources to alleviate the unique burdens of navigating pregnancy and birth as a Black woman.

### Advocacy

Despite the wide range of feelings about racial concordance in prenatal care discussed, participants expressed a strong and unanimous desire for Black healthcare provider advocacy to improve their access to mental health care. They wanted mental health advocacy specifically because of the difficulty they experience gaining access, their unique needs for mental health support around racism, and a deep belief that mental health care was fundamental to healthy pregnancies. They saw this as an important way of engaging Black patients like themselves in care:

But you need more Black advocacy in that field [mental health], which means that some people [Black providers and patients] are going to have to get up, and stand up, and rise up.

…I would say that looking and listening and being a part of your own struggle. I would say you need strong Black advocators…

Though some disclosed doubts about the ability of Black mental health providers to care for Black patients solely on the basis of racial concordance, most participants also expressed a desire to see more “Black advocacy in the field of mental health.” Participants used the term “Black advocacy” to describe Black providers and patients working together to advocate for better mental health access. There was also a sentiment that patients needed to start “advocating for themselves” and “being a part of [their] own struggle” in addition to having providers who were willing to advocate for them. Participants expressed that having Black advocates in mental health care would be particularly meaningful to them given the racism that affects their mental health in way they thought Black providers were uniquely equipped to understand.

## Discussion

This study was designed to determined how group prenatal care can be reimagined to fit the particular needs of Black women given the body of literature that suggests that Black women derive a unique benefit from prenatal care. The literature around the unmet mental health needs of Black pregnant women, and the interconnection between racism and the mental health needs of Black women, led us to solicit the opinions of our participants on the role of mental health and racially concordant care in group prenatal care. This work has only become more important as the negative effects of COVID-19 has been disproportionally experienced by Black people. We chose to have self-identified Black facilitators for this focus group to foster candid conversations on racism in healthcare.

We found that within a group of pregnant and recently postpartum Black women in San Francisco, group prenatal care was a welcomed concept. We also found that women emphasized comprehensive, accessible and race-conscious behavioral health services as a highly needed and desirable component to patient-centered perinatal care. This desire for care that directly addresses structural racism has also been reported elsewhere (Chambers et al., 2021). Our participants’ focus on mental health needs is not surprising given recent data in a cohort similar to ours that Black peripartum women experience multidimensional chronic stressors rooted in racism that may affect their mental health and birth outcomes (Chambers et al., 2020). A prospective cohort study of 244 Black women receiving prenatal care in Boston found that self-reported racial discrimination was associated with perinatal depression, and that interventions that involved talking to providers about discrimination may reduce risk of depressive symptoms (Ertel et al., 2012). Moreover, their desire for providers with an understanding of structural racism and white supremacy is consistent with other studies that have reported the primacy of racially conscious care in a heterogeneous sample of Black patients, some of whom did and did not want racially-concordant care (Cuevas et al., 2016).

Participants identified several current challenges to receiving peripartum mental health care, including insufficient access and advocacy, and provider distrust. The desire for culturally competent care irrespective of provider race is reflected in many recent trainings to that end that currently exist and have been studied and reviewed. A recent systematic review of the literature on cultural competency looked at seven studies and found that cultural competency training significantly improved patient satisfaction in six of the seven studies reviewed (Govere & Govere, 2016). However, only one of the studies reviewed included Black patients, and none of the trainings focused specifically on preparing physicians to discuss racism and its effect of health with patients. This problem was highlighted in another review of cultural humility and cultural competency trainings that identified short workshops without comprehensive antiracism skills training as insufficient to prepare providers who have never experienced racial trauma to address it in their patients (Murray-García et al., 2014). These data, in combination with the women from our study who wanted “anything but White” providers after past racist treatment, are concerning that our evolution from tolerance to cultural competence to cultural humility and now antiracism curricula has still yet to adequately restore trust in the healthcare community lost by centuries of mistreatment of Black women (Owens, 2017; Washington, 2006).

The wide range of feelings that women had about racially concordant care shows the richness of our data despite our small sample size. Black women have voiced strong desires for racially-concordant prenatal care in another recent qualitative study of 22 Black women in a similar population (Altman et al., 2020). Additionally, even some of the women who said they did not want racially concordant care expressed desires for providers who had personal understanding of their experiences when probed. One such participant reported that she wanted racial concordance for their children. These nuanced responses show the complexity of patient feelings about racial concordance.

Interestingly, the patient-centered and racially-conscious advocacy that our participants asked for in our study can be found in a study that interviewed seven Black providers giving racially-concordant care in a Minnesota birthing center (Almanza et al., 2019). All of the providers in this study reported that doing such racial justice work was a large part of the reason they chose their careers. This data may suggest that the interests of Black patients and Black providers are aligned, and that Black prenatal providers may have insight into the needs of Black patients that we have yet to capitalize on in obstetric practice and antiracism efforts.

The generalizability of these data is limited by selection bias, as participation in the focus group was limited to those who elected to participate from the BIH client list. The small sample size and geographic constraints also limit generalizability. Additionally, as the pool of potential participants was restricted to women receiving services from BIH, the voices represented in the focus group may represent women who are motivated to seek out comprehensive prenatal care. Finally, we used the word “women” in our recruitment and may have limited the gender diversity of our sample by doing so. However, our sample is strengthened by UCSF’s partnership with BIH, a community organization that has earned the trust of Black pregnant women with intersecting stigmatized identities in San Francisco. This may be why our team was able to get results consistent with other literature in the field despite the small sample size. Moreover, the diversity of opinions found in our focus group suggests that our sample was large enough to explore a myriad of perceptions, opinions, and beliefs of Black pregnant women in ways useful for future research and intervention, as is the intention of qualitative studies.

More work is needed to illuminate the ways in which group prenatal care can be tailored to best help Black pregnant and postpartum women. Since it is clear that better mental health access is needed based on our data, we recommend that it be included as a foundational aspect of any group prenatal care curriculum designed for Black women. Since racism plays such a large role in the mental health of Black peripartum women, we recommend that facilitators for Black group prenatal care receive training on how to facilitate using critical race theory/anti-racism lens, and get recommended training (Knight et al., 2019). In California, where our study took place, mental health services for government insured pregnant patients only covered until 3 months post-partum (*Expanding Postpartum Medicaid Coverage | The Henry J. Kaiser Family Foundation*, n.d.). This coverage is insufficient to meet the mental health burden highlighted in our study, therefore we also recommend provider advocacy for accessible mental health services. Though our patients have a wide range of opinions on the role of racial concordance in their care that also warrants further investigation, they are single minded in their desire for Black provider led mental health advocacy. As health equity advocates, we must also recommend this improvement.
